# Inferring transmission risk of respiratory viral infection from the viral load kinetics of SARS-CoV-2, England, 2020 to 2021 and influenza A virus, Hong Kong, 2008 to 2012

**DOI:** 10.2807/1560-7917.ES.2025.30.6.2400234

**Published:** 2025-02-13

**Authors:** Jakob Jonnerby, Joe Fenn, Seran Hakki, Jie Zhou, Kieran J Madon, Aleksandra Koycheva, Sean Nevin, Rhia Kundu, Michael A Crone, Timesh D Pillay, Shazaad Ahmad, Nieves Derqui, Emily Conibear, Robert Varro, Constanta Luca, Paul S Freemont, Graham P Taylor, Maria Zambon, Wendy S Barclay, Jake Dunning, Neil M Ferguson, Benjamin J Cowling, Ajit Lalvani, on behalf of the ATACCC Study Investigators

**Affiliations:** 1 NIHR Health Protection Research Unit in Respiratory Infections, National Heart and Lung Institute, Imperial College London, London, United Kingdom; 2 Section of Virology, Department of Infectious Disease, Imperial College London, London, United Kingdom; 3 NIHR Health Protection Research Unit in Emerging and Zoonotic Infections, University of Oxford, Oxford, United Kingdom; 4 Section of Structural and Synthetic Biology, Department of Infectious Disease, Imperial College London, London, United Kingdom; 5 UK Dementia Research Institute Centre for Care Research and Technology, Imperial College London, London, United Kingdom; 6 London Biofoundry, Imperial College Translation and Innovation Hub, London, United Kingdom; 7 UK Health Security Agency, London, United Kingdom; 8 NIHR Health Protection Research Unit Modelling and Health Economics, MRC Centre for Global Infectious Disease Analysis, Jameel Institute, Imperial College London, London, United Kingdom; 9 Department of Virology, Manchester Medical Microbiology Partnership, Manchester Foundation Trust, Manchester Academic Health Sciences Centre, Manchester, United Kingdom; 10 World Health Organization Collaborating Centre for Infectious Disease Epidemiology and Control, School of Public Health, Li Ka Shing Faculty of Medicine, The University of Hong Kong, Hong Kong Special Administrative Region, China; 11 Laboratory of Data Discovery for Health (D24H) Limited, Hong Kong Science and Technology Park, Hong Kong Special Administrative Region, China; 12 The members of the ATACCC Study Investigators are acknowledged at the end of the article; * These authors contributed equally to this work.

**Keywords:** SARS-CoV-2, influenza A, viral load kinetics, infectiousness, transmission risk, decline time, decline rate, clearance time

## Abstract

**Background:**

Infectiousness of respiratory viral infections is quantified as plaque forming units (PFU), requiring resource-intensive viral culture that is not routinely performed. We hypothesised that RNA viral load (VL) decline time (e-folding time) in people might serve as an alternative marker of infectiousness.

**Aim:**

This study’s objective was to evaluate the association of RNA** **VL decline time with RNA and PFU** **VL area under the curve (AUC) and transmission risk for SARS-CoV-2 and influenza A virus.

**Methods:**

In SARS-CoV-2 and influenza A virus community cohorts, viral RNA was quantified by reverse transcription quantitative PCR in serial upper respiratory tract (URT)-samples collected within households after an initial household-member tested positive for one virus. We evaluated correlations between RNA** **VL decline time and RNA and PFU-VL AUC. Associations between VL decline time and transmission risk in index-contact pairs were assessed.

**Results:**

In SARS-CoV-2 cases, we observed positive correlations between RNA** **VL decline time and RNA and PFU** **VL AUC with posterior probabilities 1 and 0.96 respectively. In influenza A cases a positive correlation between RNA** **VL decline time and RNA** **VL AUC was observed, with posterior probability of 0.87. Index case VL decline times one standard deviation above the cohort-mean showed a relative increase in secondary attack rates of 39% (95%** **credible interval (CrI):** **−6.9** **to** **95%) for SARS-CoV-2 and 25% (95% CrI:** **−11 to 71%) for influenza A virus.

**Conclusion:**

We identify VL decline time as a potential marker of infectiousness and transmission risk for SARS-CoV-2 and influenza A virus. Early ascertainment of VL kinetics as part of surveillance of new viruses or variants could inform public health decision making.

**Key public health message**

**What did you want to address in this study and why?**

Understanding respiratory viruses’ infectiousness is important to model infection dynamics in a population and the viral load (amount of virus in a person’s body) is relevant to infectiousness. As genetic material (e.g. RNA) of viruses can be used to detect them, we investigated if the viral RNA decline time (time for the RNA viral load to reduce by a factor e of ca 2.72) could be used as a marker for SARS-CoV-2 or influenza A virus infectiousness.

**What have we learnt from this study?**

RNA viral decline time, found by quantifying viral RNA in consecutive respiratory samples, correlated with the amount of virus shed in individuals infected with SARS-CoV-2 (in the United Kingdom) and influenza A virus (in Hong Kong, Special Administrative Region, China). In 69 SARS-CoV-2 and 153 influenza A cases found as first infected in a household (index cases), a slower viral load decline time was predictive of a higher transmission risk.

**What are the implications of your findings for public health?**

As the RNA viral load decline time correlates with infectiousness and transmission risk, and is easier to quantify by PCR using viral RNA in serial respiratory samples than the infectious viral load obtained by methods such as quantitative viral culture, it could be used in studies investigating the infectiousness of new respiratory viruses or variants.

## Introduction

RNA viral load (VL) kinetics of respiratory viral infections reflect the balance between viral replication and the host immune response; they are used to inform population-level viral infection models [1,2] and as an outcome measure in clinical trials of antivirals [3]. Understanding whether RNA VL kinetics predict transmission is therefore relevant for public health decision making.

The inoculum dose of live virions, measured using in-vitro viral culture plaque assays and quantified as plaque forming units (PFU) [4], associates with infection risk in challenge models [5,6] and PFU VL is recognised as a key determinant of individual infectiousness. However, viral culture to quantify PFU is not possible for routine clinical or surveillance sampling, which instead relies on quantifying RNA by reverse-transcription quantitative (RT-q)PCR. The duration of severe acute respiratory syndrome coronavirus 2 (SARS-CoV-2) culture-positive viral shedding is usually short, with a study undertaken between 13 September 2020, and 28 October 2021 in the United Kingdom (UK) finding a median infectious period of 5 days [7], and RNA detected outside this time-window does not necessarily indicate infectiousness. Further, given that the same study found that the ratio between SARS-CoV-2 RNA VL and PFU varies 100-fold over the course of infection [7], the timing of sample collection relative to the time of infection likely affects whether RNA VL at a given time point reflects infectiousness. While symptom onset timing may be used to estimate time of infection, recall bias and the prevalence of asymptomatic infections limits the accuracy and availability of such information. In the present paper, we hypothesise that the area under the curve (AUC) of the RNA VL trajectory associates with AUC of the PFU trajectory, making it a potential marker of infectiousness. However, AUC is challenging to measure, as serial upper respiratory tract (URT)-sampling is required from infection onset.

**Figure 1:**
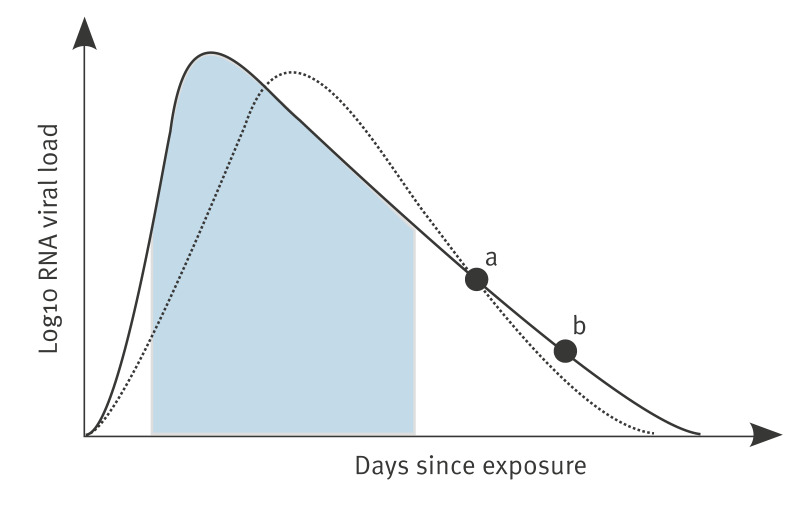
Illustration of the advantage of using two samples to estimate the viral load area under the curve

Single time point sampling is insufficient to accurately infer the earlier VL trajectory (Figure 1), but VL decline time, the time for the VL to decrease by a factor e of ca 2.72 when using the natural logarithmic base e (the choice of logarithmic base is arbitrary), can be used to infer the earlier VL trajectory as it correlates negatively with the VL growth time [8] and is less sensitive to the time of sample collection [3]. We therefore hypothesised that RNA VL decline time correlates with RNA and PFU VL AUC, even when RNA VL decline time is estimated using samples collected outside the narrow window of infectious viral shedding.

A single URT sample (point a) is compatible with multiple viral trajectories, illustrated by the solid and dashed lines. Moreover, as the sample was collected outside the window of infectious viral shedding (represented by the shaded blue area), it is not representative of the actual level of exposure to contacts. Calculating the viral decline time using two URT samples (points a and b) provides a better estimate of the true viral trajectory and the VL AUC and therefore of the infectiousness. The evolution with time, of the Log10 of RNA viral load is represented; the principle applies with another logarithmic base (in the current study a natural logarithmic base is used).

We further leveraged two globally unique prospective household transmission studies of SARS-CoV-2 and influenza A virus with serial URT-sampling of index cases from infection onset, where the accurate estimation of secondary attack rates (SAR) enabled us to test whether RNA VL decline time also correlates with transmission risk. Previous analyses of these cohorts found no correlations between the maximum measured VL and SAR [9,10], or between the VL decline time and peak VL [8].

## Methods

Both SARS-CoV-2 and influenza A virus studies recruited households after an initial (index) household-member tested RT-PCR-positive on a URT-swab. Participants who were recruited in the UK and in Hong Kong Special Administrative Region, China, provided serial URT-samples regardless of presence or absence of symptoms.

**Figure 2:**
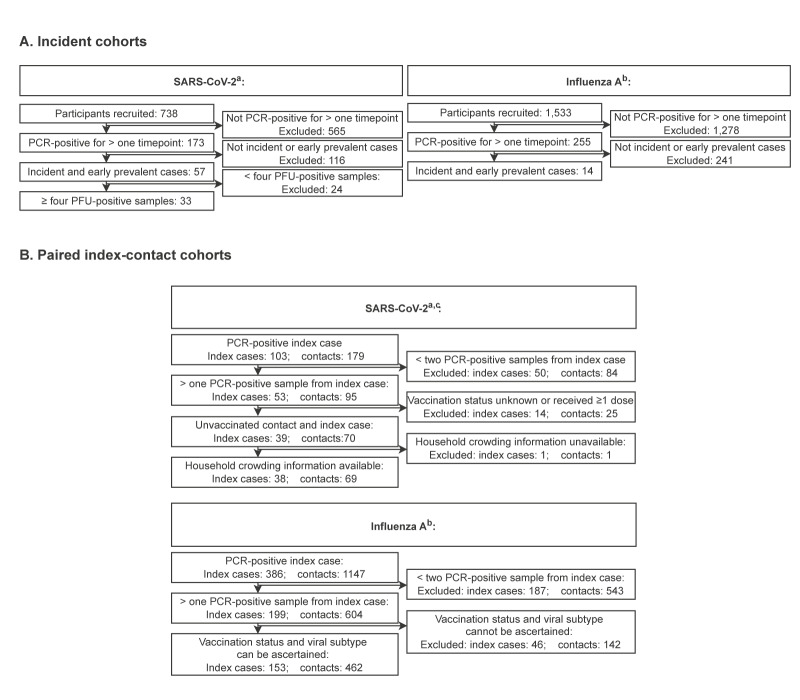
Flowchart of the recruited SARS-CoV-2 and influenza A virus incident cohorts and paired index-contact cohorts, with SARS-CoV-2 cases recruited in England, United Kingdom, 2020–2021, and influenza A cases in Hong Kong Special Administrative Region, China, 2008–2012 (n** **=** **2,541 participants recruited)

Participants in the SARS-CoV-2 cohorts, recruited to the ATACCC and INSTINCT studies between 2020 and 2021 in London and Manchester, UK [8,11], were infected with pre-alpha, alpha, or delta variants. URT-swabs and blood samples were collected by healthcare professionals from INSTINCT participants at enrolment and on days 7, 14 and 28, with a self-swabbed sample provided on day 4. The ATACCC participants provided daily self-swabbed URT-samples. Vaccinated participants were defined as those who had received their second COVID-19 vaccination ≥ 14 days before index symptom onset; none had received a third dose (Figure 2, Table).

PCR: polymerase chain reaction; PFU: plaque forming units; SARS-CoV-2: severe acute respiratory syndrome coronavirus 2.

^a^ More detail on this cohort can be found in [7].

^b^ More detail on this cohort can be found in [10]. Twelve contacts among the paired index-contact cohorts were also part of the incident cohorts.

^c^ More detail on this cohort can be found in [11].

(A) Incident cases were used to analyse correlations between viral decline time and AUC and peak VL. ‘Incident’ refers both to participants who were PCR-negative on the day of study enrolment and became PCR-positive during the study, and to prevalent cases who were PCR-positive from the day of recruitment, but in whom the growth phase and peak VL were captured. (B) The paired index-contact cohorts were used to test associations between index cases’ VL decline time and the probability of their contacts becoming PCR-positive.

**Table:** Demographics of the SARS-CoV-2 and influenza A virus index-contact cohorts, with SARS-CoV-2 cases recruited in England, United Kingdom, 2020–2021, and influenza A cases in Hong Kong Special Administrative Region, China, 2008–2012 (n** **=** **722 participants)

Variable		Overall, n	Overall, %	Transmitted^a^, n.	Transmitted^a^, %	p^b^
**Influenza A**						
**Influenza A index cases ** **(n = 153)**						
Index cases		153	100	40	100	NA
Age in years	Median (IQR)	10 (6 to 15)		10 (6 to 11)		NA
	0–19	124	81.0	35	87.5	0.08
	20–34	13	8.5	0	0.0	
	≥ 35	16	10.5	5	12.5	
Sex (binary variable)	Female	66	43.1	19	47.5	0.64
	Male	87	56.9	21	52.5	
Comorbidities^c^	No	135	88.2	37	92.5	0.49
	Yes	18	11.8	3	7.5	
Subtype	Pandemic A(H1N1)	12	7.8	2	5.0	0.59
	Seasonal A(H1N1)	88	57.5	22	55.0	
	Seasonal A(H3N2)	53	34.6	16	40.0	
Vaccination > 14 days prior	Unvaccinated	130	85.0	33	82.5	0.8
	Vaccinated	23	15.0	7	17.5	
Antivirals	No	95	62.1	25	62.5	1
	Yes	58	37.9	15	37.5	
Ethnicity (%)	Non-white	NC	NC	NC	NC	NA
	White	NC	NC	NC	NC	
People per bedroom	0 to 1	NC	NC	NC	NC	NA
	> 1 to 2	NC	NC	NC	NC	
	> 2 to 3	NC	NC	NC	NC	
Number of contacts	1	0	0.0	0	0.0	0.5
	2	51	33.3	10	25.0	
	3	61	39.9	16	40.0	
	4	32	20.9	10	25.0	
	5	5	3.3	2	5.0	
	6	4	2.6	2	5.0	
**Influenza A contacts (n = 462)**						
Contacts		462	100	48	100	NA
Age in years	Median (IQR)	38 (28 to 46)		36 (23 to 46)		NA
	0–19	87	18.8	12	25.0	0.49
	20–34	116	25.1	12	25.0	
	≥ 35	259	56.1	24	50.0	
Sex (binary variable)	Female	295	63.9	31	64.6	1
	Male	167	36.1	17	35.4	
Comorbidities^c^	No	378	81.8	40	83.3	0.93
	Yes	84	18.2	8	16.7	
Subtype	Pandemic A (H1N1)	31	6.7	3	6.2	0.71
	Seasonal A (H1N1)	273	59.1	26	54.2	
	Seasonal A (H3N2)	158	34.2	19	39.6	
Vaccination in most recent autumn before recruitment	Unvaccinated	409	88.5	42	87.5	1
	Vaccinated	53	11.5	6	12.5	
Ethnicity	Non-white	NC	NC	NC	NC	NA
	White	NC	NC	NC	NC	
People per bedroom	0 to 1	NC	NC	NC	NC	NA
	> 1 to 2	NC	NC	NC	NC	
	> 2 to 3	NC	NC	NC	NC	
**SARS-CoV-2**						
**SARS-CoV-2 index cases (n = 38)**						
Index cases		38	100	20	100	NA
Age in years	Median (IQR)	30 (23 to 43)		41 (34 to 46)		NA
	0–19	5	13.2	1	5.0	0.004
	20–34	16	42.1	5	25.0	
	≥ 35	17	44.7	14	70.0	
Sex (binary variable)	Female	17	44.7	9	45.0	1
	Male	21	55.3	11	55.0	
Comorbidities^c^	No	30	78.9	17	85.0	0.571
	Yes	8	21.1	3	15.0	
Variant	Pre-alpha	11	28.9	3	15.0	0.135
	Alpha	24	63.2	15	75.0	
	Delta	3	7.9	2	10	
Vaccination > 14 days prior	Unvaccinated	38	100	20	100	1
	Vaccinated	0	0	0	0.0	
Antivirals	No	38	100	20	100	1
	Yes	0	0	0	0.0	
Ethnicity	Non-white	6	15.8	3	15.0	1
	White	32	84.2	17	85.0	
People per bedroom	0 to 1	19	50.0	9	45.0	0.398
	> 1 to 2	18	47.4	11	55.0	
	> 2 to 3	1	2.6	0	0.0	
Number of contacts	1	21	55.3	10	50.0	0.25
	2	5	13.2	4	20.0	
	3	10	26.3	4	20.0	
	4	2	5.3	2	10.0	
**SARS-CoV-2 serology cohort index cases (n = 25)** ^d^						
Index cases		25	100	14	100	NA
Age in years	Median (IQR)	37 (23 to 43)		42 (38 to 47)		NA
	0–19	3	12.0	0	0.0	0.007
	20–34	9	36.0	3	21.4	
	≥ 35	13	52.0	11	78.6	
Sex (binary variable)	Female	12	48.0	6	42.9	0.86
	Male	13	52.0	8	57.1	
Comorbidities^c^	No	20	80.0	11	78.6	1
	Yes	5	20.0	3	21.4	
Variant	Pre-alpha	9	36.0	3	21.4	0.20
	Alpha	16	64.0	11	78.6	
	Delta	0	0.0	0	0.0	
Vaccination > 14 days prior	Unvaccinated	25	100	14	100	1
	Vaccinated	0	0.0	0	0.0	
Antivirals	No	25	100	14	100	1
	Yes	0	0.0	0	0.0	
Ethnicity	Non-white	5	20.0	2	14.3	0.76
	White	20	80.0	12	85.7	
People per bedroom	0 to 1	15	60.0	7	50.0	0.46
	> 1 to 2	10	40.0	7	50.0	
	> 2 to 3	0	0.0	0	0.0	
Number of contacts	1	14	56.0	8	57.1	0.20
	2	5	20.0	4	28.6	
	3	5	20.0	1	7.1	
	4	1	4.0	1	7.1	
**SARS-CoV-2 contacts (n = 69)**						
Contacts		69	100	28	100	NA
Age in years	Median (IQR)	25 (16 to 41)		27 (15 to 40)		NA
	0–19	21	30.4	10	35.7	0.5
	20–34	23	33.3	7	25.0	
	≥ 35	25	36.2	11	39.3	
Sex (binary variable)	Female	36	52.2	15	53.6	1
	Male	33	47.8	13	46.4	
Comorbidities^c^	No	60	87.0	26	92.9	0.4
	Yes	9	13.0	2	7.1	
Variant	Pre-alpha	20	29.0	4	14.3	0.1
	Alpha	45	65.2	22	78.6	
	Delta	4	5.8	2	7.1	
Vaccination > 14 days prior	Unvaccinated	69	100	28	100	1
	Vaccinated	0	0	0	0	
Ethnicity	Non-white	15	21.7	6	21.4	1
	White	54	78.3	22	78.6	
People per bedroom	0 to 1	32	46.4	11	39.3	0.4
	> 1 to 2	36	52.2	17	60.7	
	> 2 to 3	1	1.4	0	0.0	
**SARS-CoV-2, serology cohort contacts (** **n = 43** **)** ^d^						
Contacts		43	100	18	100	NA
Age in years	Median (IQR)	28 (20 to 49)		36 (19 to 43)		NA
	0–19	11	25.6	5	27.8	0.62
	20–34	13	30.2	4	22.2	
	≥ 35	19	44.2	9	50.0	
Sex (binary variable)	Female	20	46.5	9	50.0	0.94
	Male	23	53.5	9	50.0	
Comorbidities^c^	No	35	81.4	17	94.4	0.14
	Yes	8	18.6	1	5.6	
Variant	Pre-alpha	18	41.9	14	77.8	0.06
	Alpha	25	58.1	4	22.2	
	Delta	0	0	0	0	
Vaccination > 14 days prior	Unvaccinated	43	100	18	100	1
	Vaccinated	0	0	0	0	
Ethnicity	Non-white	10	23.3	3	16.7	0.62
	White	33	76.7	15	83.3	
People per bedroom	0 to 1	22	51.2	8	44.4	0.66
	> 1 to 2	21	48.8	10	55.6	
	> 2 to 3	0	0	0	0	

IQR: interquartile range; NA: not applicable; NC: not collected; SARS-CoV-2: severe acute respiratory syndrome coronavirus 2.

^a^ For the contacts, ‘transmitted’ refers to the contacts who got infected by the index cases, while for the index cases, ‘transmitted’ refers to the index cases who infected contacts.

^b^ In index cases the χ^2^ test was used to calculate differences between index cases who did transmit the virus, and those who did not, and in contacts between those who at some point tested PCR-positive, and those who did not.

^c^ For SARS-CoV-2 comorbidities included asthma, cancer, chronic cardiac disease, diabetes, epilepsy, high blood pressure, liver disease, rheumatologic disorder, and vasovagal syncope. No information was obtained on what comorbidities were included in the influenza virus cohort.

^d^ Index cases and contacts in the serology cohort were seronegative on the first study day.

RT-PCR results confirmed that index case participants in the influenza virus cohort, recruited between 2008 and 2012 in Hong Kong [10], were infected with H1N1 or H3N2 subtypes. Trained research staff collected URT-swabs from all participants on three occasions within a 7-day period, with no viral culture attempted. Participants were defined as vaccinated if they received influenza vaccination in the most recent autumn before recruitment, noting that influenza activity in Hong Kong occurs in the winter and also in the spring and summer in some years (Figure 2, Table) [10].

Cohorts of SARS-CoV-2 and influenza virus index-contact pairs were identified through epidemiological linkage to investigate transmission risk. For SARS-CoV-2, this included participants from both the ATACCC and INSTINCT cohorts, where whole genome sequencing carried out in 16 of 28 infected contacts confirmed that the SARS-CoV-2 variant was identical in index and contact. Households with multiple index cases were excluded from the analysis.

Some infected cases enrolled before their VL peaks; these were referred to as ‘incident cases’ and were used to investigate correlations between VL decline time and AUC.

Only incident cases from the ATACCC cohort were included in the analysis of SARS-CoV-2 VL AUC because daily sample collection in the ATACCC study enabled better estimation of VL AUC than in INSTINCT, where samples were collected less frequently. Plaque assays were carried out on 90% (547/605) of the PCR-positive samples from the 57 incident SARS-CoV-2 cases. Some samples from six of the 57 incident individuals collected in MANTACC brand of virus transport medium (VTM) were not cultured as the medium proved to be toxic to Vero E6 cells and the remaining samples could not be recovered for assay.

### Statistical analyses

We used a hierarchical Bayesian model to estimate the VL decline time and other kinetic parameters of viral trajectories of incident and index cases, as previously described [8]. In incident cases, we used a linear model to estimate the correlation between VL decline time and VL AUC, and in the identified index-contact pairs we used a beta-binomial model to estimate the association between index RNA VL decline time and transmission risk. Posterior probabilities (pp) and Bayes factors (BF = pp/(1 − pp)) were computed for associations between variables being positive; pp ≥ 0.75 (BF > 3) was interpreted as at least moderate evidence of an association. This is further detailed in the Supplementary Materials.

## Results

### Viral shedding

**Figure 3:**
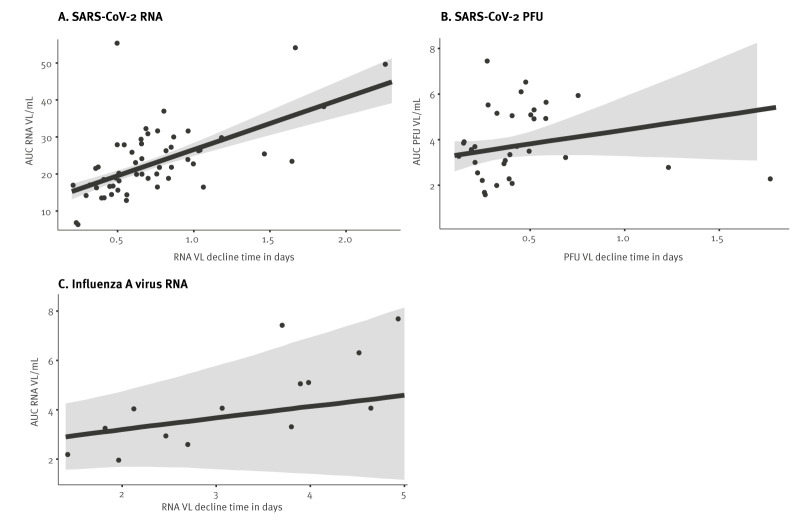
Scatter plots of the VL AUC as a function of the VL decline time for A) SARS-CoV-2 RNA (n = 57), B) SARS-CoV-2 PFU (n = 33), and C) influenza A virus RNA (n = 14), with SARS-CoV-2 cases recruited in England, United Kingdom, 2020–2021, and influenza A cases in Hong Kong Special Administrative Region, China, 2008–2012

AUC: area under the curve; PFU: plaque forming units; SARS-CoV-2: severe acute respiratory syndrome coronavirus 2; VL: viral load.

We investigated correlations between RNA VL decline time, defined as the time for the VL to decrease by a factor of e of ca 2.72, VL peak and VL AUC in incident cases. The ATACCC study enrolled 57 incident SARS-CoV-2 cases, of which 41 were PCR-negative and 16 PCR-positive on the first study day, and with 33 shedding infectious virus detectable as PFUs over at least 4 days [7]. The RNA decline times correlated with RNA AUC (pp = 1, Figure 3A) and PFU AUC (pp = 0.96); moreover, PFU decline times correlated with PFU AUC (PFU: pp = 0.83; Figure 3B); the model parameter estimates are detailed in Supplementary Tables S3 and S4. The AUCs were calculated from a power of 0.1 of the VL, i.e. VL^γ^ with γ = 0.1, as previously suggested [12], with calculations from other powers presented in Supplementary Table S5. The RNA VL AUC correlated with the PFU VL AUC (pp = 0.999); this is also detailed in Supplementary Table-S6. The time between receiving the second vaccine dose and the first study day in 23 incident vaccinated SARS-CoV-2 participants was 104 days on average (standard deviation (SD): 44 days). We found no evidence of a positive correlation between time since vaccination and RNA VL decline time (pp = 0.45).

The black points show individual incident participants, the solid lines and shaded grey areas show linear fits and 95% credible intervals.

Fourteen incident influenza cases were enrolled, all of which were PCR-positive on the first study day (Figure 2), as shown in Supplementary Table S2. RNA decline time correlated with RNA AUC (pp = 0.87), for a power of 0.1 of the VL with model parameter estimates presented in Supplementary Table S7 (Figure 3C); estimated correlations between the RNA decline time and RNA AUC for other powers figure in Supplementary Table S5.

### Accuracy of viral decline time estimates

**Figure 4:**
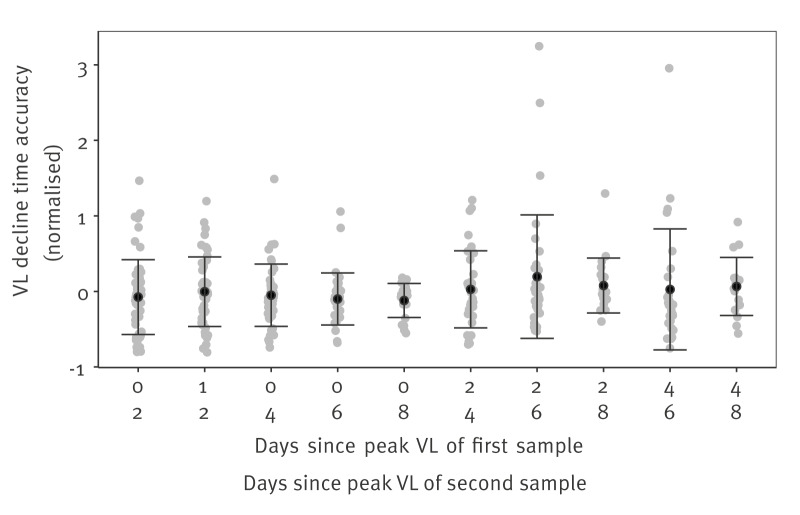
Scatter plot of the accuracy of using two samples to estimate the viral load decline time, with SARS-CoV-2 cases recruited in England, United Kingdom, 2020–2021 (n = 57)

SARS-CoV-2: severe acute respiratory syndrome coronavirus 2; VL: viral load.

We performed a sensitivity analysis using SARS-CoV-2 incident cases to assess the accuracy of using only two positive RNA VL samples to estimate the VL decline time. On average 7.5 (interquartile range (IQR): 5 to 9) positive samples were collected during the viral decline phase of each incident case. We selected two samples from different days and calculated the accuracy as the relative difference between the estimated VL decline time when using those two samples with that obtained when using all available samples. The selected days and calculated accuracy are shown in Figure 4. In general, the accuracy increased with a longer time between samples. The highest accuracy was obtained when the first sample was collected on the day of peak viral load (day 0) and the second sample 8 days later, with 75% of estimates being within ± 25% of the true value, followed by samples collected on days 2 and 8, 4 and 8, and 0 and 6 respectively with 70%, 65%, and 56% of estimates being within ± 25% of the true value; the calculated accuracy on different sample collection days is shown in Supplementary Table S8.

The top and bottom rows of the x-axis show the days of collection, relative to the day of peak VL, for the first and second sample used. The y-axis shows the relative difference between the VL decline time estimated using two samples with that obtained when using all available samples. Grey points represent individual SARS-CoV-2 incident cases, black points the mean accuracy, and error bars the standard deviation.

### Transmission risk

**Figure 5:**
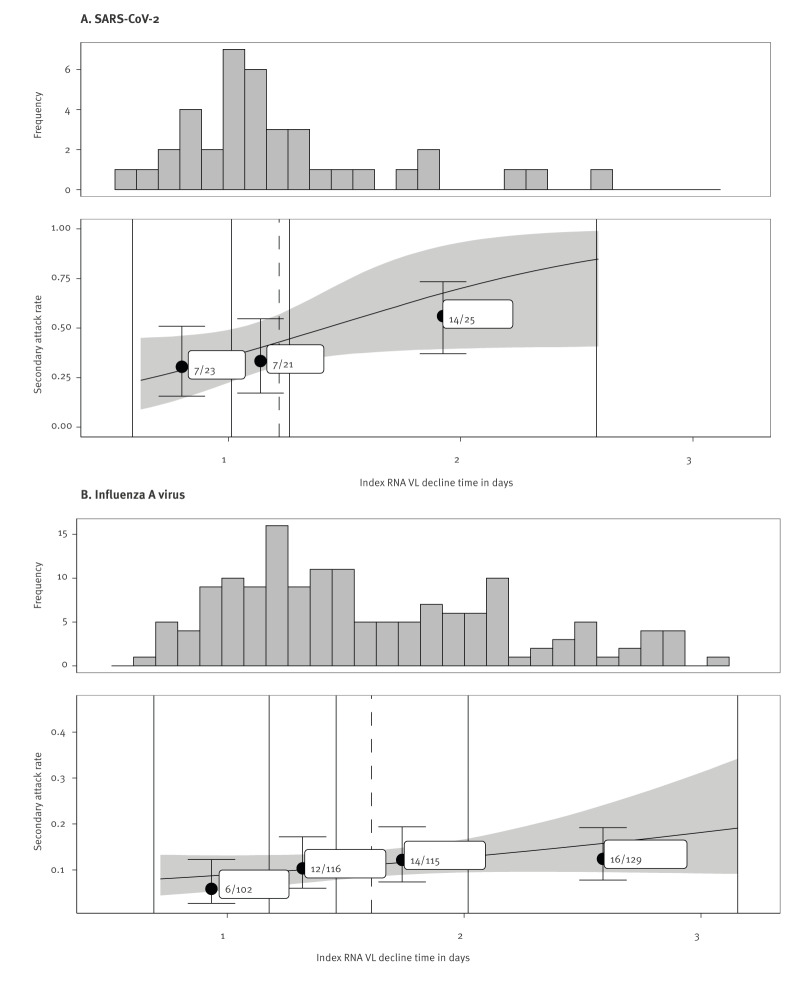
(Top) Frequency distributions of the viral RNA decline times in index cases and (bottom) the association between index viral RNA decline time and SAR for (A) SARS-CoV-2 (n = 38) and (B) influenza A virus (n = 153), with SARS-CoV-2 cases recruited in England, United Kingdom, 2020–2021, and influenza A cases in Hong Kong Special Administrative Region, China, 2008–2012

SAR: secondary attack rate; SARS-CoV-2: severe acute respiratory coronavirus 2; VL: viral load.

Having established that RNA VL decline time correlates with RNA and PFU VL AUC, we next investigated the hypothesis that VL decline time may therefore correlate with transmission risk. A total of 38 SARS-CoV-2 index-cases were linked with 69 household contacts, including only unvaccinated participants as few were vaccinated at enrolment. From SARS-CoV-2 index-cases an average of 3.2 (IQR: 2 to 3) positive samples, with viral loads > 20 copies/mL, were collected over on average 6.8 (IQR: 4 to 7) days. Index VL decline times were on average 1.22 days (SD: 0.46 days; Figure 5A), with model parameter estimates presented in Supplementary Table S9.

For the two bottom graphs in panels A and B, the solid vertical lines divide the cohort into intervals containing a similar number of index-cases. The numbers, points and error bars indicate respectively the infected over total number of contacts, mean SAR and 95% binomial confidence intervals in each interval. The dashed vertical lines indicate the mean viral decline times of index cases in the cohort. The median and 95% credible interval of the correlation between RNA decline time and SAR obtained using the beta-binomial distribution are indicated by the solid lines and the shaded grey areas.

The SAR was 41% (28/69) among SARS-CoV-2 contacts, with longer index case VL decline times associated with a higher SAR using a beta-binomial model (pp = 0.95). To estimate the effect size, we compared the SAR when index VL decline time was equal to the cohort mean, with that of index VL decline times one SD greater than the cohort mean. In the latter case, we estimated an, on average, 39% (95% credible interval (CrI): −6.9% to 95%) relative increase in the SAR, corresponding to an absolute SAR increase of 16% (95% CrI: −2.8% to 36%) (Figure 5A); individual data points are shown in Supplementary Figure S1A and the model parameter estimates in Supplementary Table S10. Among index cases who infected all contacts, 10 had just one contact, two had two contacts, and one had three contacts in total.

Adjusted analyses using the beta-binomial model supported these associations, but with insufficient sample sizes to compare effect sizes at different covariate values (pp = 0.82); the model parameter estimates are presented in Supplementary Table S11. Confounders included variant, age, comorbidities, and people per bedroom. We used leave-one-out cross-validation to test for potential model overfitting: the performance was found to be similar for the adjusted and unadjusted models. Small differences were observed between the models, where the unadjusted model performed better than the adjusted model; the cross-validated model performance is shown in Supplementary Table S12.

The associations remained when estimating RNA VL decline time using just two URT samples randomly selected during the decline phase, with an unadjusted effect size of 35% (95% CrI: −23% to 89%) (pp = 0.90), estimated using the beta-binomial model as a relative increase in the SAR. Using all available positive URT samples from index cases to estimate the RNA viral decline time, positive associations with SAR were also detected using unadjusted logistic models, with an effect size of 50% (95% CrI: 2.9% to 117%), estimated as a relative increase in the SAR (pp = 0.98; model parameter estimates in Supplementary Table S13), and after covariate adjustment (pp = 0.91; model parameter estimates in Supplementary Table S14).

Collection of blood samples allowed us to measure the presence of antibodies, indicating previous exposure or infection with SARS-CoV-2. We performed a sensitivity analysis, removing index cases (n = 6) and contacts (n = 11) with measurable antibodies on the first day and contacts where blood samples were unavailable (n = 12). Contacts with seropositive index cases (n = 3) and index cases with only seropositive contacts (n = 7) were also removed from the analysis. In 43 seronegative contacts of 25 index cases, the secondary attack rate was 42% (18/43). In an unadjusted analysis using a beta-binomial model, we found a positive correlation between the index VL decline time and SAR (pp = 0.99), corresponding to a relative effect size of 46% (95%CrI: 8% to 120%). The observed association remained after covariate adjustment (pp = 0.97).

A total of 199 influenza index-cases were linked with 604 contacts; vaccination status and viral strain was ascertained in 153 index cases and 462 contacts [10] (Figure 2). From influenza index-cases, 2.3 (IQR: 2 to 3) positive samples, with viral loads > 900 copies/mL, were collected over on average 4 (IQR: 3 to 5) days. Index VL decline times were on average 1.59 days (SD: 0.58) (Figure 5B), with model parameter estimates presented in Supplementary Table S9. The SAR was 10% (48/462) among contacts. Using a beta-binomial model, the relative effect size was 25% (95% CrI: −11% to 71%) (pp = 0.90) (Figure 5B), corresponding to a 2.8% (95% CrI: −1.3% to 7.5%) absolute increase in the SAR; individual data points are shown in Supplementary Figure S1B and model parameter estimates in Supplementary Table S10. Only two index cases infected all of their contacts, with two contacts in total each.

Adjusted analyses also found evidence for positive associations, but with insufficient sample sizes to compare the effect size of different covariate values (pp = 0.94); the model parameter estimates are presented in Supplementary Table S11. Confounders included subtype, age, comorbidities, index and contact vaccination status. We used leave-one-out cross-validation to test for potential model overfitting: the performance was found to be similar for the adjusted and unadjusted models, with the adjusted model performing better; the cross-validated model performance is shown in Supplementary Table S12.

The associations remained when estimating RNA VL decline time using just two URT samples randomly selected during the decline phase, with a relative effect size of 27% (95% CrI: −18% to 86%) (pp = 0.87). Using all available positive URT samples from index cases to estimate the RNA viral decline time, positive associations with SAR were also detected using unadjusted logistic models, with a relative effect size of 26.4% (95% CrI: −8.8% to 72%) (pp = 0.95; model parameter estimates in Supplementary Table S13) and after covariate adjustment (pp = 0.95; model parameter estimates in Supplementary Table S14).

## Discussion

Using independent cohort studies of SARS-CoV-2 and influenza A virus household transmission, we found that RNA VL decline time correlated with RNA VL AUC (reflecting overall viral shedding) for both viruses and, for SARS-CoV-2, with PFU VL AUC (reflecting overall infectious viral shedding). Furthermore, RNA VL decline time correlated with transmission risk of both viruses, identifying VL decline time as a potential marker of transmission risk. However, the 95% CrIs for the relative increase in SAR with longer VL decline times were found to be wide and include zero, indicating uncertainty in the estimated parameter values. When excluding index cases and contacts with measurable antibodies against SARS-CoV-2 on the first study day, the strength of evidence of a positive association remained despite the smaller sample size.

Interestingly, viral RNA clearance times were shown to be faster in second infections compared with first SARS-CoV-2 infections in a recent study undertaken in the United States between 11 March 2020, and 28 July 2022. Moreover, clearance times of first and subsequent infections in the same individual were correlated, suggesting an inherent link between an individual’s host response and their RNA VL decline time [13].

The association between RNA VL decline time and SAR have several potential implications for public health practice. Measuring the VL decline time in household studies and infection surveys could help determine the distribution of infectiousness in different populations, enabling for example the targeting of interventions to reduce transmission. Together with other epidemiological parameters such as the reproduction number, they can also help inform population infection dynamic models with a more realistic distribution of infectiousness to improve model accuracy. Finally, measuring the VL decline time could help identify immune markers associated with infectiousness, potentially informing new medical interventions against transmission.

Single time point URT RNA VL did not correlate with transmission risk in several previous studies [9,10,14]. In other studies, however, single time point URT RNA VL did associate with SAR of SARS-CoV-2 [15-18] and influenza A virus [19]. These latter studies generally had larger sample sizes, providing greater statistical power to detect correlations between URT RNA VL and SAR. Single time point RNA VL thus appears to be a less sensitive marker of transmission than RNA VL decline time, failing to correlate with SAR in smaller studies.

Our cohort studies were conducted in different regions, timeframes, and populations. The SARS-CoV-2 cohort mainly consisted of white British adults, whereas the influenza virus cohort primarily included Chinese individuals, with most index cases being children and contacts including children and adults. Despite differences in study designs and VL kinetics, we found a consistent relationship between VL decline time and SAR for SARS-CoV-2 and influenza A virus. The lower SAR in the influenza A virus cohort was likely due to a higher degree of pre-existing immunity to influenza virus.

Our study had limitations, including insufficient sample size to resolve differences in VL decline time or transmission risk between viral variants and age groups. We cannot exclude the possibility that some contacts were the source of infection, or became infected from outside the household, but this likely constitutes a minority of cases as epidemiological linkage confirmed the index-contact pairings [9,10]. We could not account for the impact of time since vaccination in index cases, as all SARS-CoV-2 index cases were unvaccinated and no data were available on time since vaccination for influenza A index cases. We did not collect data on time since, or variant or subtype of, prior infection. With a low number of samples in the influenza A virus incident trajectories, the computation of the AUC is likely less accurate than that of SARS-CoV-2 trajectories. The applicability of RNA VL decline time to predict, and enable prevention of, transmission on an individual level is limited as a proportion of transmissions will occur early during infection, before RNA VL decline time can be measured. Instead, our findings are more suitable as a tool to improve understanding of infection dynamics at a population level. Furthermore, only the relative difference in infectiousness between individuals can be estimated using the VL decline time alone; household transmission studies are required to determine the absolute relation between VL decline time and SAR, which is specific to each virus. Larger independent studies would help to confirm our findings.

## Conclusions

In conclusion, RNA VL decline time, quantified by serial URT-samples collected during the viral decline phase is a potential marker of infectiousness and transmission risk for the two most important respiratory viruses globally. Our findings suggest that VL decline time can potentially inform our understanding of population-level respiratory viral infection dynamics. Larger transmission studies could be used to establish the value of estimating VL decline time to inform risk assessment and public health decision making early in an outbreak of a new respiratory virus or variant of an existing virus. This could be achieved by promptly commissioning household transmission studies with serial URT-sampling as part of pandemic preparedness toolkits.

## Supplementary Data

171000 Supplementary Material
