# Supplementary material for: Inferring transmission risk of respiratory viral infection from the viral load kinetics of SARS-CoV-2, England, 2020 to 2021 and influenza A virus, Hong Kong, 2008 to 2012
Source: Euro Surveill. 2025 Feb 13;30(6):2400234. doi: 10.2807/1560-7917.ES.2025.30.6.2400234 (PMC11914967; doi:10.2807/1560-7917.ES.2025.30.6.2400234)
Supplement: Supplementary Material [file 2400234_SupplementaryMaterial.pdf]

Supplementary Material

This supplementary material is hosted by Eurosurveillance as supporting information alongside the article "Inferring transmission risk of respiratory viral infection from the viral load kinetics of SARS-CoV-2, England, 2020 to 2021 and influenza A virus, Hong Kong, 2008 to 2012" on behalf of the authors, who remain responsible for the accuracy and appropriateness of the content. The same standards for ethics, copyright, attributions and permissions as for the article apply. Supplements are not edited by Eurosurveillance and the journal is not responsible for the maintenance of any links or email addresses provided therein.

Supplementary figures

Figure S1 Distribution of the viral RNA decline time and its association with SAR for SARS-CoV-2 and influenza A

Supplementary tables

Table S1 Dates of recruitment of SARS-CoV-2 index cases and their contacts
Table S2 The demographic characteristics of influenza A and SARS-CoV-2 incident cases
Table S3 Estimated parameters of the SARS-CoV-2 RNA VL model
Table S4 Estimated parameters of the SARS-CoV-2 PFU VL model
Table S5. Estimated correlation between the viral load decline time and AUC viral load for different powers of the viral load.
Table S6. Estimated correlation between the SARS-CoV-2 RNA AUC viral load and PFU AUC viral load
Table S7 Estimated parameters of the Influenza A VL model
Table S8 Accuracy of estimating the SARS-CoV-2 VL decline time using two samples when compared to using all samples.
Table S9 Estimated parameters of the SARS-CoV-2 and Influenza A index VL decline time model
Table S10 Estimated unadjusted beta-binomial model parameters of the association between the SARS-CoV-2 and Influenza A index viral decline time and secondary transmission.
Table S11 Estimated adjusted beta-binomial model parameters of the association between the SARS-CoV-2 and Influenza A index viral decline time and secondary transmission.
Table S12 Model evaluation using Leave One Out Cross Validation (LOO-CV)
Table S13 Estimated unadjusted logistic model parameters of the association between the SARS-CoV-2 index viral decline time and secondary transmission.
Table S14 Estimated adjusted logistic model parameters of the association between the SARS-CoV-2 and Influenza A index viral decline time and secondary transmission.

Supplementary Methods

Study recruitment. **Page 2**
PCR assays. **Page 2**
Plaque assays. **Page 2**
Viral whole genome sequencing for lineage assignments. **Page 3**
Modelling viral kinetics. **Page 7**
Modelling the association between kinetic variables **Page 8**
Beta-binomial model of the association between viral decline time and secondary attack rate **Page 9**
Modelling the association between viral decline time and secondary attack rate using a logistic model **Page 10**

Study recruitment

Close contacts of newly PCR-positive SARS-CoV-2 index cases were notified to the UK contract tracing system (National Health Service Test and Trace) and invited to participate if they were (i) 5 years and older, (ii) within 5 days of the index case's symptom onset and (iii) could provide informed consent. Recruited contacts self-performed combined nose (anterior nares) and throat swabs (hereafter denoted upper respiratory tract [URT] swabs) at home after detailed instruction by study nurses, for up to 20 consecutive days.

ATACCC enrolment spanned two separate time periods: ATACCC1 enrolled 393 contacts from 327 households from 13th September 2020 to 31st March 2021 during the SARS-CoV-2 pre-alpha and alpha variant waves while ATACCC2 enrolled 345 contacts from 215 households from 24th May, 2021, to October 28th, 2021, during the delta variant wave. During enrolment, demographic information was collected using questionnaires. Vaccination records were obtained from GP records with consent. Unvaccinated cases were defined as those who had not received any COVID-19 vaccination prior to index symptom onset, our proxy for exposure. Fully vaccinated cases were defined as those who had received their second COVID-19 vaccination ≥14 days prior to index symptom onset. Cases who received only one dose of vaccine prior to index symptom onset were excluded. Due to the timing of our study recruitment respective to the UK vaccine rollout, none of our vaccinated contacts had more than two vaccinations. Variant ascertainment was through a combination of methods, with 25 index cases and 16 contacts ascertained through whole genome sequencing (WGS), 1 index case through SGTF, 13 index cases and 45 contacts through date of recruitment, and 9 contacts who were assumed to have the same variant as their index case. Participants recruited before 5 November, 2020, were assumed to be infected with the pre-alpha variant, those recruited between 14 December, 2020, and 31 March, 2021 were assumed to be infected with the alpha variant, and those recruited after 15 May, 2021, were assumed to be infected with the delta variant.

PCR assays

For the INSTINCT study, viral RNA was extracted via the innuPREP Virus TS RNA 2.0 Kit (Analytik Jena) on the CyBio Felix (Analytik Jena), following the manufacturer's instructions but without the addition of carrier RNA. A multiplex quantitative real-time PCR was used for the E gene and RNase P assay, and a singleplex reaction was performed for the S gene dropout. Reactions were run with the TaqMan Fast Virus 1-Step Master Mix (ThermoFisher Scientific), with the addition of a SARS-COV-2 positive control and negative control.

Plaque assays

For the ATACCC study, 3ml of viral transport media (VTM) (2 brands: Copan Universal Transport Medium System, Copan Diagnostics; VTM2, MANTACC) from PCR-confirmed SARS-CoV-2-positive samples were thawed from -80°C, and used in plaque assays. African green monkey kidney (VeroE6) cells expressing human angiotensin-converting enzyme 2 (ACE2) and transmembrane protease serine 2 precursor (TMPRSS2) were kindly provided by MRC-University of Glasgow Centre for Virus Research (CVR), Glasgow[1]. The cells were maintained in Dulbecco's modified Eagle's medium (DMEM; Gibco), 10% fetal calf serum (FCS; Gibco), 1 mg/mL Geneticin (Gibco), 0.2 mg/mL Hygromycin B (Invitrogen). VeroE6 cell monolayers grown in 12-well plates were washed with Phosphate-buffered saline (PBS) and infected with a 10-fold dilution series of 200µl VTM samples. Plates were incubated at 37°C in 5% CO2 for 1 hour. At 1 hour post-infection, the inoculum was removed and cell monolayers were overlaid with DMEM supplemented with 0.2% w/v bovine serum albumin (Gibco), 0.16% w/v NaHCO3 (Gibco), 10 mM HEPES (Invitrogen), 2 mM L-Gutamine (Gibco), 1X P/S and 0.6% Avicel (Gibco). Following the 72 hours incubation period, the methylcellulose overlay was removed and cells were fixed with 4% paraformaldehyde at room temperature for 20 minutes. The paraformaldehyde was removed, and wells were stained for 1 hour with 1ml of 0.05% crystal violet in 20% methanol. Plates were washed with tap water then dried. Plaques were counted at the dilution in which there were 5-50 plaque forming units (PFUs). The limit of detection of the assay was less than 10 plaque forming units. Samples which were collected in MANTACC brand VTM were not cultured as the media proved to be toxic to Vero E6 cells and the remaining samples could not be recovered for assay.

Viral whole genome sequencing for lineage assignments

For WGS performed for the SARS-CoV-2 pre-alpha, alpha and delta variants, automated RNA extraction was performed using a CyBio FeliX (Analytik Jena) and the innuPREP Virus TS RNA Kit 2.0 (Analytik Jena) according to the manufacturer's instructions, with a sample volume of 200µl, without carrier RNA and with an elution volume of 50µl. RT-qPCR was repeated using an in-house protocol[2]. cDNA synthesis was then performed using the LunaScript RT SuperMix Kit (NEB) according to the manufacturer's instructions with a total reaction volume of 20 µl and extracted sample volume of 5µl. Libraries were generated using the EasySeq™ RT-PCR SARS CoV-2 (novel coronavirus) Whole Genome Sequencing kit v2 or v3 (Nimagen) according to the manufacturer's instructions. Samples were then pooled and purified with AMPure XP (Beckman Coulter) magnetic beads. Suitable quality of libraries was confirmed using a Tapestation (Agilent) and concentrations were measured using the Qubit 1x dsDNA High Sensitivity Assay Kit (Thermofisher Scientific) and Qubit 4 Fluorometer (ThermoFisher Scientific). Pooled libraries were then diluted down to 55pM. The final pool was then run on an iSeq 100 (Illumina) with a total of 322 cycles (151 bp paired reads and 10 bp indices). Generated fastq files were processed using the EasySeq variant pipeline (v0.6.0 for kit version 2 or v0.8.1 for kit version 3)[3] which is a Nextflow[4] pipeline that uses fastp[5], BWA MEM[6], SAMtools[7], BCFtools[7], LoFreq[8], mosdepth[9], BEDtools[9], SnpEff[10] and MultiQC[11] to QC, trim and assemble the reads (using reference sequence NC_045512.2) and then generate a consensus sequence and variant report before assigning a PANGO lineage[12] pangolin (v3.1.17, lineages version 2021-12-06)[13]. In the analyses presented here, both genomically-probable and genomically-confirmed cases were included. Pre-alpha status was assigned to cases where alpha infection, and infections caused by other variants of concern or variants under investigation had been excluded.

Table S1 dates of recruitment of SARS-CoV-2 index cases and their contacts

Participant type	Time period	Numbers recruited
Index cases (INSTINCT)	1 August 2020 to 23 March 2021	94
Index cases (ATACCC)	13 September 2020 to 31 March 2021	1
Index cases (ATACCC)	24 May 2021 to 28 October 2021	8
Contacts (INSTINCT)	1 August 2020 to 23 March 2021	245
Contacts (ATACCC)	13 September 2020 to 31 March 2021	393
Contacts (ATACCC)	24 May 2021 to 28 October 2021	345
Households (INSTINCT)	1 August 2020 to 23 March 2021	94
Households (ATACCC)	13 September 2020 to 31 March 2021	327
Households (ATACCC)	24 May 2021 to 28 October 2021	215

Table S2 The demographic characteristics of influenza A and SARS-CoV-2 incident cases. A) Influenza A incident cases. No statistical differences were calculated between different subtypes due to the low number of participants. **B) SARS-CoV-2 incident cases infected with delta, alpha and pre-alpha strains and vaccinated cases infected with the delta variant.** χ^2 tests were performed to determine differences for each characteristic between unvaccinated and fully vaccinated cases.

A)

		**Cases**	
		**n.**	**%**
N		14	100
Age (median [IQR])		6.50 [5.25, 7.00]	
	(0,19]	13	92.9
	(19,35]	0	0.0
	(35,100]	1	7.1
Sex	Female	5	35.7
	Male	9	64.3
Chronic disease	No	13	92.9
	Yes	1	7.1
Subtype	Unsubtypable	2	14.3
	Seasonal A (H1N1)	9	64.3
	Seasonal A (H3N2)	3	21.4
Vaccination status	Unvaccinated	13	92.9
	Vaccinated	1	7.1
Antiviral treatment	No	8	57.1
	Yes	6	42.9

B)

		All contacts		Pre-alpha		Alpha		Delta un-vaccinated		All unvaccinated		Delta vaccinated		
		n.	%	n.	%	n.	%	n.	%	n.	%	n.	%	P
	N	33	100	9	100	4	100	5	100	18	100	15	100	
Age	Median [IQR]	41.00 [27.00, 46.00]		35.00 [27.00, 46.00]		47.00 [40.25, 52.50]		13.00 [12.00, 15.00]		28.00 [17.00, 45.75]		43.00 [40.00, 47.00]		0.06
	<18	6	18.2	0	0	0	0	5	100	5	27.8	1	6.7	
	18-50	22	66.7	8	88.9	3	75	0	0	11	61.1	11	73.3	
	51-65	5	15.2	1	11.1	1	25	0	0	2	11.1	3	20	
	65+	0	0	0	0	0	0	0	0	0	0	0	0	
Sex	F	16	48.5	4	44.4	2	50	1	20	7	38.9	9	60	0.39
	M	17	51.5	5	55.6	2	50	4	80	11	61.1	6	40	
Ethnicity	Non-White	3	9.1	0	0	0	0	0	0	0	0	3	20	0.17
	White	30	90.9	9	100	4	100	5	100	18	100	12	80	
Co-morbidities[a] or pregnancy	No	26	78.8	7	77.8	3	75	4	80	14	77.8	12	80	1
	Yes	7	21.2	2	22.2	1	25	1	20	4	22.2	3	20	
Smoker	Current	2	6.1	0	0	0	0	0	0	0	0	2	13.3	0.19
	Former	5	15.2	2	22.2	0	0	0	0	2	11.1	3	20	
	No	26	78.8	7	77	4	100	5	100	16	88.9	10	66.7	
Exposure [b]	House-hold	30	88.5	6	66.7	4	100	5	100	15	88.5	15	100	0.29
	Non-house-	3	9.1	3	33.3	0	0	0	0	3	16.7	0	0	

[a] Health conditions included asthma (n=4), chronic obstructive pulmonary disease (n=1), hepatitis B (n=1), moderate liver disease (n=1), second trimester of pregnancy (n=1).

[b] Index symptom onset was used as a proxy for exposure.

Modelling viral kinetics

In order to estimate the kinetic parameters of the individual RNA and PFU trajectories in incident cases, we used a phenomenological Bayesian hierarchical model of the following form

$$v(\tau) = v_{max} \frac{(1/a + 1/b)}{e^{-\frac{(\tau-\tau_{max})}{a}}/b + e^{\frac{(\tau-\tau_{max})}{b}}/a} \quad (1)$$

where $v(\tau)$ is the RNA copies/ml or PFU/ml concentration at time τ, v_{max} its maximum value which occurs at time $\tau = \tau_{max}$, a the growth time and b the decline time. Exponential growth occurs for times $\tau \ll \tau_{max}$, and exponential decline for times $\tau \gg \tau_{max}$. In index cases, where RNA samples where only collected during the decline phase, a simpler exponential decay model was used instead:

$$v(\tau) = v_{max} e^{-(\tau-\tau_{max})/b}$$

The index VL decline time, therefore, refers to the parameter *b* in the equation above. The model specification allows for test failures, i.e. that the sample was not drawn from a normal distribution with mean given by the equation for $v(\tau)$ in equation (1), with probability p, and for censored data below the detection limit of the assays. The log-likelihood of observing a value x at time τ in the trajectory is given by:

$$l(x) = \log[p\, n(x,\, x_0,\, \sigma_0) + (1-p) n(x,\, \log(v),\, \sigma_v)],$$

where $n(x,\, x_0,\, \sigma_0)$ represents the normal probability density function with mean x_0 and standard deviation σ_0 at point x. False positive and negative test results were assumed to be normally distributed, with x_0 determining the ratio between them, $x_0 = 0$ implying a ratio of 1:1.. When the observations are below the limit of detection, $x = 0$, we use the cumulative density function of the normal distribution to account for censored data

$$l(x=0) = \log[p\mathrm{N}(0,\, x_0,\, \sigma_0) + (1-p)\mathrm{N}(0,\, \log(v),\, \sigma_v)],$$

where $\mathrm{N}(x,\, \mu, \sigma)$ is the cumulative density function of the normal distribution with mean μ and standard deviation σ. Since the three parameters a_j, b_j, and $v_{max,\,j}$ for each participant j are positive numbers they were expressed in exponential form: $\theta_{1,j} = \log(v_{max,\,j})$, $\theta_{2,j} = \log(a_j)$, $\theta_{3,j} = \log(b_j)$. The ATACCC participants were grouped by vaccination status, allowing the kinetic parameters of each group to be represented with a different group mean, whereas for influenza A no adjustments were made due to the low number of incident cases (N=14). Using a non-centred parametrisation to allow for a more efficient exploration of the parameter space, the parameters of each participant were expressed as

$$\theta_{i,\,j} = \mu_{i,\,k(j)} + \delta_i\, z_{i,\,j},$$

where $k = \{1,2\}$, with 1 being unvaccinated, and 2 being vaccinated participants, and each participant j mapped to one of the two groups via $k(j)$. In the sensitivity analysis based on a smaller cohort consisting of participants with at least 5 positive PFU samples, we fitted a single group ($k = 1$) in order to compare the mean viral RNA and PFU kinetic parameters. Deviations from the mean were modelled using a multiplier δ_i times $z_{i,\,j}$, with prior

$$z_i \sim n(0,\, C),$$

where z_i is a two-dimensional vector with elements $z_i = \{z_{1,\,i},\, z_{2,\,i}\}$, $n(0,\, C)$ a two-dimensional multivariate normal distribution, and C a correlation matrix with prior $lkj\_corr\_cholesky(1)$, using the Lewandowski-Kurowicka-Joe (LKJ) prior with Cholesky factor representation. Similar to Singanayagam *et al*, we used weakly informative priors and performed prior predictive checks to ensure that the selected priors were consistent with previously published viral trajectories[14]. $\mu_1 \sim n(15,\, 15)$ (for RNA trajectories), $\mu_1 \sim n(12,\, 12)$ (for PFU trajectories), $\mu_2 \sim n(0.8,\, 0.48)$, $\mu_3 \sim n(2,\, 1.8)$, $\delta_1 \sim n_{>0}(0,\, 10)$, $\delta_2 \sim n_{>0}(0,\, 0.64)$, $\delta_3 \sim n_{>0}(0,\, 1.8)$, with $n_{>0}$ indicating normal distributions truncated at zero from below. The priors were chosen to allow for a broad range of values of the kinetic parameters and were compatible with the trajectories of previously investigated infecting variants.

Time τ was measured in days and defined on a participant-specific basis such that $\tau = 0$ was the first study day, and the time of peak was given the prior probability $\tau_{max} \sim n(5,5)$.

The error probability p was fitted on a log scale such that $(-\log p) \sim n_{\geq 0}(5,2)$ (i.e. giving a relatively uninformative truncated [above at 1] lognormal prior for p with mean 0.04 and standard deviation 0.12) while x_0 was given a prior of $n(0,1)$ (giving a 1:1 ratio of false positives and negatives). Both σ_0 and σ_v were given the relatively uninformative prior of $n_{\geq 1}(3,3)$, where the minimum lower bound of 1 prevented Markov Chain Monte Carlo (MCMC) divergence issues associated with exploring very low values of these measurement precision related parameters. In order to avoid artificially steep gradients when the viral load crossed the assay limit of detection (LoD) thresholds, the LoD was subtracted from all data points in the modelling (such that the lowest data points would be at zero) and was afterwards added to the estimated viral trajectories. The limits of detection were given as 3.4 and 2.3 in natural logarithm units for the RT-PCR and plaque assays, respectively.
Using the individually fitted trajectories we calculated the within-sample means of the kinetic parameters as

$$\log(v_{max,k}) = \Sigma_{j \in \{j \mid k(j)=k\}} \theta_{1,j} \,/\, n_k \,,$$
$$a_k = \Sigma_{j \in \{j \mid k(j)=k\}} \exp(\theta_{2,j}) \,/\, n_k \,,$$
$$b_k = \Sigma_{j \in \{j \mid k(j)=k\}} \exp(\theta_{3,j}) \,/\, n_k \,.$$

Modelling was conducted in R version 4.1.2[15] using the package RStan[16] to fit the models using Hamiltonian MCMC methods. For each model fit, 8 MCMC chains of 8,000 iterations each were undertaken. The first 3,000 iterations of each chain were used during the burn-in phase, and each chain was thinned by half, giving a total of 20,000 posterior samples for each model. Standard Stan diagnostics were used to confirm convergence, mixing and adequate effective sample sizes.

Modelling the association between kinetic variables

A Bayesian hierarchical mixture model was used to calculate the association between the AUC viral load shedding and the viral load growth and decline times, controlling for vaccination, infecting variant, and demographic confounders. The model is given by, for SARS-CoV-2,

$$\hat{y} \sim \mathrm{n}(\beta\hat{x} + \gamma V + Z, \sigma),$$

and for influenza A,

$$\hat{y} \sim \mathrm{n}(\beta\hat{x}, \sigma),$$

where $\hat{y}$ is the AUC viral load, $\hat{x}$ the viral decline time with parameter β, γ the effect of vaccination, V a vaccine indicator variable ($V = 0$ for unvaccinated and $V = 1$ for vaccinated participants), Z a vector to account for different variants, $Z = \{z_1, z_2, z_3\}$, with z_1 used for the pre-alpha, z_2 for alpha, and z_3 for the delta infecting variants for SARS-CoV-2. No adjustments were done for the Influenza A cohort due to the low number of samples. We assumed $\hat{y}$ was normally distributed, with standard deviation σ. The uncertainty of the modelled decline time was accounted for by assuming that the true viral decline time $\hat{x}$ is unknown, but that the calculated mean of the modelled distribution ($\bar{x}$) follows a normal distribution with mean $\hat{x}$, and the modelled standard deviation σ_x as the width of the distribution:

$$\bar{x} \sim \mathrm{n}(\hat{x}, \sigma_x),$$

Similarly, it is assumed that the true outcome parameter $\hat{y}$ is unknown, and that the mean $\bar{y}$ of the calculated distribution is normally distributed with mean $\hat{y}$:

$$\bar{y} \sim \mathrm{n}(\hat{y}, \sigma_y).$$

The regression parameters were given priors $\hat{x} \sim \mathrm{n}(mean(\bar{x}), 4 * sd(\bar{x}))$, $\hat{y} \sim \mathrm{n}(mean(\bar{y}), 4 * sd(\bar{y})),)$, $\beta \sim n(0, 2)$, $\sigma \sim \mathrm{n}(0, 2)$, $\gamma \sim \mathrm{n}(0, 2)$. The variant-dependent offset parameter was model using a non-centred hierarchical representation:

$$z_i = \mu_i + z_{SD,i}\mathrm{n}(0, 1),$$

where μ_i is the mean of the distribution and $z_{SD,i}$ its standard deviation, which in turn are assumed to be normally distributed with:

$$\mu_i \sim \mathrm{n}(\chi, \omega)$$
$$z_{SD,i} \sim \mathrm{n}_{>0.3}(\rho, \varphi).$$

The distribution of the standard deviation was assumed to be bounded at 0.3 from below to avoid divergence issues. The hyperparameters were similarly given the priors $\chi \sim \mathrm{n}\left(0.5 * mean(\bar{x}), 0.5 * \mathrm{sd}(\bar{x})\right)$, $\omega \sim \mathrm{n}_{>0.3}(0, 4 * \mathrm{sd}(\bar{x}))$, $\rho \sim \mathrm{n}\left(0, 4 * \mathrm{sd}(\bar{x})\right)$, $\varphi \sim \mathrm{n}_{>0.3}(0, 4 * \mathrm{sd}(\bar{x}))$.

Beta-binomial model of the association between viral decline time and secondary attack rate

The association between the RNA viral load decline time of index cases and the number of secondary infections was investigated using a Bayesian beta-binomial regression model for SARS-CoV-2,

$$S_i \sim \mathrm{BetaBin}(N_{Ci}, \alpha, \theta),$$
$$\alpha = \theta p/(1-p)$$
$$p = \mathrm{logit}(\xi\hat{x} + Z + \gamma + \kappa K + \delta D)$$

and for Influenza A,

$$S_i^{vacc} \sim \mathrm{BetaBin}(N_{Ci}^{vacc}, \alpha^{vacc}, \theta^{vacc}),$$
$$\alpha^{vacc} = \theta^{vacc}p^{vacc}/(1-p^{vacc})$$
$$p^{vacc} = \mathrm{logit}(\upsilon^{con} + \upsilon^{ind}V^{ind} + \xi\hat{x} + Z + \gamma + \delta D + \lambda A)$$

$$S_i^{unvacc} \sim \mathrm{BetaBin}(N_{Ci}^{unvacc}, \alpha^{unvacc}, \theta^{unvacc}),$$
$$\alpha^{unvacc} = \theta^{unvacc}p^{unvacc}/(1-p^{unvacc})$$
$$p^{unvacc} = \mathrm{logit}(\upsilon^{ind}V^{ind} + \xi\hat{x} + Z + \gamma + \delta D + \lambda A)$$

where S_i is the number of secondary infections from the index, p the mean probability of transmission, N_{Ci} the number of recruited contacts of the index case, θ a shape parameter in the Beta distribution, $\hat{x}$ the viral RNA decline time with parameter ξ, Z a vector to account for different variants, $Z = \{z_1, z_2, z_3\}$, with z_1 used for the pre-alpha, z_2 for alpha, and z_3 for the delta infecting variants for SARS-CoV-2, and seasonal H1N1, seasonal H3N2, and pandemic H1N1 respectively for Influenza A, γ a categorical parameter for the index age, κ a parameter for household crowding, with K the number of household members per bedroom, D an indicator variable for the presence of comorbidities with parameter δ. Since no participants in the SARS-CoV-2 paired index-contact cohort were vaccinated, vaccination status was not included as a parameter. However, some participants in the Influenza A paired index-contact cohort were vaccinated which was controlled for by fitting separate beta-binomial models for vaccinated and unvaccinated contacts with joint parameters. In the Influenza A model, υ^{con} and υ^{ind} are parameters that adjust for differences in the secondary attack rate between vaccinated and unvaccinated contacts and index cases, respectively, V^{ind} an indicator variable for index vaccination status, , and λ a parameter for the effect of antiviral treatment of the index case on transmission, with A an indicator variable of antiviral treatment.

As described in the section above, the uncertainty of the modelled decline time was accounted for by assuming that the true viral decline time $\hat{x}$ is unknown, but that the calculated mean of the modelled distribution $(\bar{x})$ follows a normal distribution with mean $\hat{x}$, and the modelled standard deviation σ_x being the width of the distribution:

$$\bar{x} \sim \mathrm{n}(\hat{x}, \sigma_x),$$

Using the z-scored decline time, the regression parameters were given priors $\hat{x} \sim \mathrm{n}(0,2)$, $\hat{y} \sim \mathrm{n}(0,2)$, $\xi \sim n(0,2)$, $\sigma \sim \mathrm{n}(0,2)$, $\gamma \sim \mathrm{n}(0,2)$, $\theta \sim \exp(1)$, $\delta \sim \mathrm{n}(0,2)$, $\lambda \sim \mathrm{n}(0,2)$. The same priors were given to vaccinated (vacc) and unvaccinated (unvacc) contacts. The variant-dependent offset parameter was model using a non-centred hierarchical representation:

$$z_i = \mu_i + z_{SD,i}\mathrm{n}(0,1),$$

where μ_i is the mean of the distribution and $z_{SD,i}$ its standard deviation, which in turn are assumed to be normally distributed with:

$$\mu_i \sim \mathrm{n}(\chi, \omega)$$
$$z_{SD,i} \sim \mathrm{n}_{>0.3}(\rho, \varphi).$$

The distribution of the standard deviation was assumed to be bounded at 0.3 from below to avoid divergence issues. The hyperparameters were similarly given the priors $\chi \sim \mathrm{n}(0,2)$, $\omega \sim \mathrm{n}_{>0.3}(0,2)$, $\rho \sim \mathrm{n}(0,2)$, $\varphi \sim \mathrm{n}_{>0.3}(0,2)$.

Modelling the association between viral decline time and secondary attack rate using a logistic model

In the logistic model, the outcome for each contact could take on the values $S_i = \{0,1\}$, with 0 for non-infected and 1 for infected, defined as having received at least one positive PCR test during the duration of the study. The model is described as, for SARS-CoV-2:

$$S_i \sim \mathrm{Bernoulli}(p),$$
$$p = \mathrm{logit}(\xi\hat{x} + Z + \gamma^{con} + \gamma^{ind} + \kappa K + \delta^{con}D^{con} + \delta^{ind}D^{ind}),$$

and for influenza A:

$$S_i \sim \mathrm{Bernoulli}(p),$$
$$p = \mathrm{logit}(\xi\hat{x} + Z + \gamma^{con} + \gamma^{ind} + v^{con}V^{con} + v^{ind}V^{ind} + + \delta^{con}D^{con} + \delta^{ind}D^{ind} + \lambda A)$$

The parameters were given priors $\gamma \sim \mathrm{n}(0,2)$, $v \sim \mathrm{n}(0,2)$, $\delta \sim \mathrm{n}(0,2)$ for index cases (ind) or contacts (con), $\kappa \sim \mathrm{n}(0,2)$ + $\lambda \sim \mathrm{n}(0,2)$. The modelling assumptions and choice of priors was otherwise same as for the beta-binomial model, described above.

Modelling was conducted in R version 4.1.2[15] using the package RStan[16] to fit the models using Hamiltonian MCMC methods. For each model fit, 4 MCMC chains of 5,000 iterations each were undertaken. The first 2,000 iterations of each chain were used during the burn-in phase, giving a total of 12,000 posterior samples for each model. Standard Stan diagnostics were used to confirm convergence, mixing and adequate effective sample sizes.

(A)

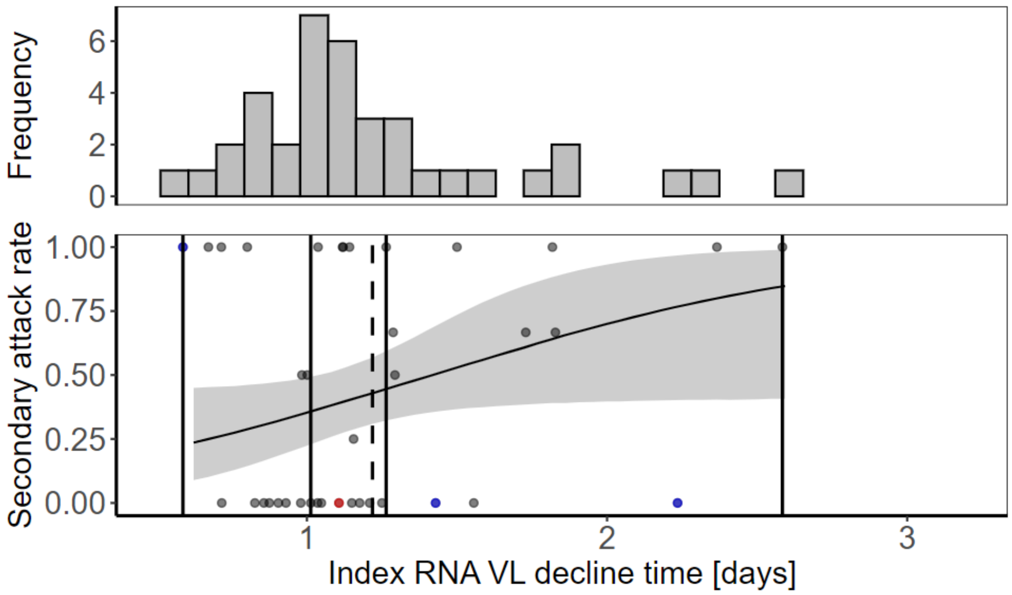

(B)

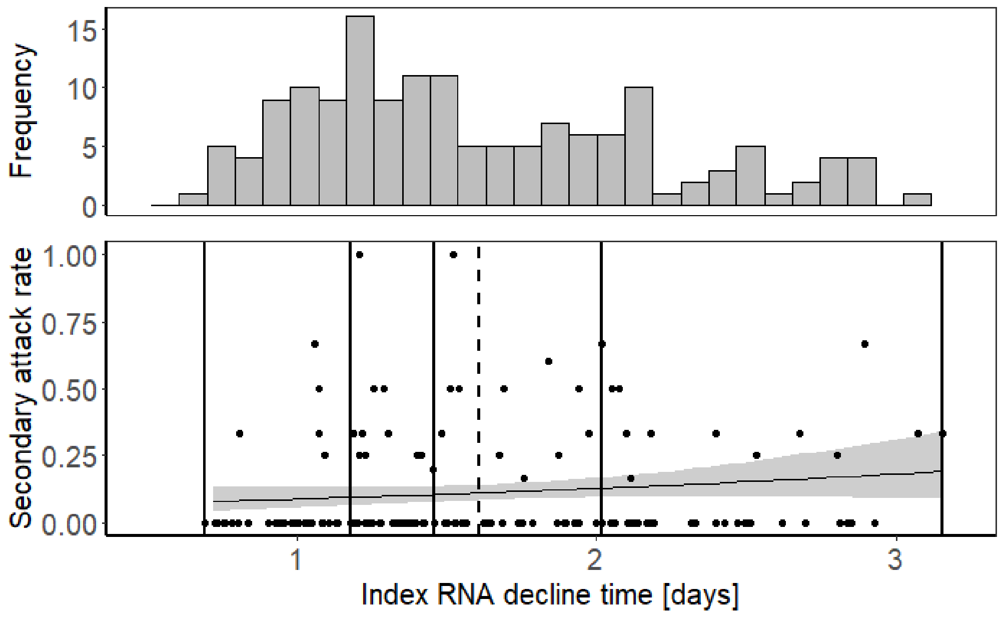

Figure S1 Distribution of the viral RNA decline time and its association with SAR for (A) SARS-CoV-2 and (B) influenza A. The solid vertical lines divide the cohort into intervals containing a similar number of index-cases. The points indicate the SAR for each index case. The dashed line indicates the mean viral decline time of index cases in the cohort. The median and 95% credible interval obtained using a beta-binomial distribution is indicated by the solid lines and the grey shaded areas. Blue and red points indicate samples with pareto-k diagnostic values >0.7 in the unadjusted and adjusted models, respectively.

Table S3 estimated parameters of the SARS-CoV-2 RNA VL model

Parameter	Parameter description	Median (95% CrI)	$\hat{R}$	n_{eff}
μ_{11}	Log max VL, unvaccinated	6.51 (6.17, 6.83)	1.01	5822
μ_{12}	Log max VL, vaccinated	6.96 (6.59, 7.31)	1.01	5586
μ_{21}	Log growth time. unvaccinated	-1.37 (-1.70, -1.01)	1.01	2987
μ_{22}	Log growth time, vaccinated	-1.36 (-1.70, -0.96)	1	3755
μ_{31}	Log decline time, unvaccinated	-0.37 (-0.59, -0.17)	1.01	1764
μ_{32}	Log decline time, vaccinated	-0.71 (-0.95, -0.48)	1.01	3245
δ_1	Log max VL deviation multiplier	1.85 (1.46, 2.39)	1.01	1922
δ_2	Growth time deviation multiplier	0.91 (0.71, 1.19)	1.03	204
δ_3	Decline time deviation multiplier	0.54 (0.43, 0.69)	1.04	86
c_{12}	Log max VL – growth time correlation	-0.17 (-0.46, 0.15)	1.01	8150
c_{13}	Log max VL – decline time correlation	0.12 (-0.20, 0.42)	1.03	225
c_{23}	Growth time – decline time correlation	-0.36 (-0.61, -0.06)	1.04	73
σ_v	VL variance	1.60 (1.48, 1.70)	1.05	57
p	Probability of false negative	0.06 (0.04, 0.08)	1.14	18
x_0	Mean of error distribution	0.75 (-2.56, 3.20)	1.08	30
σ_0	Standard deviation of error distribution	7.63 (5.95, 10.14)	1.12	20

Table S4 estimated parameters of the SARS-CoV-2 PFU VL model

Parameter	Parameter description	Median (95% CrI)	$\hat{R}$	n_{eff}
μ_{11}	Log max VL, unvaccinated	2.71 (2.10, 3.28)	1.01	5822
μ_{12}	Log max VL, vaccinated	3.66 (3.02, 4.26)	1.01	5586
μ_{21}	Log growth time. unvaccinated	-1.18 (-1.61, -0.67)	1.01	2987
μ_{22}	Log growth time, vaccinated	-1.41 (-1.82, -0.94)	1	3755
μ_{31}	Log decline time, unvaccinated	-0.90 (-1.31, -0.52)	1.01	1764
μ_{32}	Log decline time, vaccinated	-1.28 (-1.70, -0.87)	1.01	3245
δ_1	Log max VL deviation multiplier	3.11 (2.47, 3.99)	1.01	1922
δ_2	Growth time deviation multiplier	0.96 (0.70, 1.36)	1.03	204
δ_3	Decline time deviation multiplier	0.83 (0.65, 1.09)	1.04	86
c_{12}	Log max VL – growth time correlation	-0.49 (-0.74, -0.10)	1.01	8150
c_{13}	Log max VL – decline time correlation	-0.09 (-0.46, 0.29)	1.03	225
c_{23}	Growth time – decline time correlation	-0.50 (-0.75, -0.16)	1.04	73
σ_v	VL variance	1.10 (1.00, 1.24)	1.05	57
p	Probability of false negative	0.04 (0.02, 0.06)	1.14	18
x_0	Mean of error distribution	0.24 (-1.17, 1.99)	1.08	30
σ_0	Standard deviation of error distribution	2.85 (1.57, 5.28)	1.12	20

Table S5. Estimated correlation between the viral load decline time and AUC viral load for different powers of the viral load. Correlations calculated for SARS-CoV-2 RNA VL, PFU VL, and Influenza A VL. Shown are the posterior probabilities of a positive association and effect size, calculated as the mean increase in the AUC in units of standard deviations, when increasing the decline time by one standard deviation. The AUC was calculated by first calculating the power γ of the VL, i.e. VL^{γ}, for different values of γ = 0.1, 0.2, 0.3 and 0.5.

SARS-CoV-2 Power of RNA VL (N=57)	Posterior probability (pp) positive correlation	Mean effect size (95% CrI)
0.1	1	0.59 (0.41, 0.79)
0.2	1	0.49 (0.28, 0.72)
0.3	1	0.36 (0.12, 0.59)
0.5	0.91	0.13 (-0.058, 0.31)
SARS-CoV-2 Power of PFU VL (N=33)	-	-
0.1	0.83	0.2 (-0.2, 0.65)
0.2	0.78	0.15 (-0.23, 0.57)
0.3	0.72	0.12 (-0.26, 0.55)
0.5	0.63	0.055 (-0.26, 0.4)
Influenza A Power of RNA VL (N=14)	-	-
0.1	0.87	0.31 (-0.22, 0.86)
0.2	0.92	0.25 (-0.1, 0.61)
0.3	0.9	0.21 (-0.11, 0.54)
0.5	0.82	0.15 (-0.16, 0.45)

Table S6. Estimated correlation between the SARS-CoV-2 RNA AUC viral load and PFU AUC viral load. Shown are the posterior probabilities of a positive association and effect size, calculated as the mean increase in the AUC in units of standard deviations, when increasing the decline time by one standard deviation.

SARS-CoV-2 Power of RNA VL (N=33)	SARS-CoV-2 Power of PFU VL (N=33)	Posterior probability (pp) positive correlation	Mean effect size (95% CrI)
0.1	0.1	0.999	0.34 (0.11, 0.62)

Table S7 estimated parameters of the Influenza A VL model

Parameter	Parameter description	Median (95% CrI)	$\hat{R}$	n_{eff}
μ_{11}	Log max VL	1.61 (1.29, 1.92)	1	180
μ_{12}	Log growth time	0.48 (-0.24, 1.46)	1.01	140
μ_{13}	Log decline time	1.17 (0.41, 2.15)	1	155
δ_1	Log max VL deviation multiplier	0.81 (0.21, 1.70)	1	245
δ_2	Growth time deviation multiplier	0.43 (0.02, 1.44)	1	243
δ_3	Decline time deviation multiplier	0.76 (0.03, 1.94)	1.01	90
c_{12}	Log max VL – growth time correlation	0.06 (-0.85, 0.82)	1.01	138
c_{13}	Log max VL – decline time correlation	0.01 (-0.67, 0.85)	1	155
c_{23}	Growth time – decline time correlation	0.20 (-0.78, 0.93)	1	85
σ_v	VL variance	1.03 (1.00, 1.19)	1	152
p	Probability of false negative	0.00 (0.00, 0.04)	1	306
x_0	Mean of error distribution	0.40 (-6.71, 6.02)	1	330
σ_0	Standard deviation of error distribution	3.94 (1.17, 9.02)	1.01	293

Table S8 Accuracy of estimating the SARS-CoV-2 VL decline time using two samples when compared to using all samples. Each row is the accuracy when using samples on the days indicated by the two leftmost columns, with the mean and standard deviation columns are calculated from N samples indicated on the corresponding rows. Also indicated are the % of samples that were accurate within ±25% of the true value, and the number of trajectories that were excluded because the 2nd sample was higher than the 1st, which meant that the VL decline time could not be estimated, which happened in 4.1% (25/570) instances.

First sample, days since peak VL	Second sample, days since peak VL	Mean	SD	% Samples within 25% of true value	N	Excluded because 2nd sample > 1st sample
0	2	-0.08	0.50	45	56	0
0	4	-0.05	0.41	53	45	0
0	6	-0.10	0.34	56	34	0
0	8	-0.12	0.23	75	20	0
1	2	-0.003	0.46	40	50	5
2	4	0.03	0.51	45	33	12
2	6	0.20	0.82	52	33	1
2	8	0.08	0.36	70	20	0
4	6	0.03	0.80	27	26	5
4	8	0.07	0.38	65	17	2

Table S9 estimated parameters of the SARS-CoV-2 and Influenza A index VL decline time model

**Parameter**	**Parameter description**	**Median (95% CrI)**	$\hat{R}$	n_{eff}
SARS-CoV-2				
μ_1	Log growth time	5.09 (4.87, 5.31)	1	4481
μ_2	Log decline time	-0.11 (-0.18, -0.03)	1.01	3476
δ_1	Log max VL deviation multiplier	4.28 (3.93, 4.68)	1	7863
δ_2	Decline time deviation multiplier	0.54 (0.49, 0.61)	1	6513
c_{12}	Log max VL – decline time correlation	-0.54 (-0.63, -0.43)	1.01	2841
σ_v	VL variance	1.48 (1.41, 1.56)	1	7246
p	Probability of false negative	0.05 (0.04, 0.07)	1	5276
x_0	Mean of error distribution	5.64 (4.78, 6.49)	1	11054
σ_0	Standard deviation of error distribution	3.80 (3.27, 4.49)	1.01	1527
Influenza A				
μ_1	Log growth time	1.79 (1.71, 1.86)	1	4135
μ_2	Log decline time	0.41 (0.34, 0.50)	1	4590
δ_1	Log max VL deviation multiplier	1.06 (0.93, 1.22)	1	2656
δ_2	Decline time deviation multiplier	0.45 (0.38, 0.53)	1	2674
c_{12}	Log max VL – decline time correlation	-0.26 (-0.43, -0.06)	1	4251
σ_v	VL variance	1.00 (1.00, 1.01)	1	7462
p	Probability of false negative	0.00 (0.00, 0.00)	1	7315
x_0	Mean of error distribution	0.01 (-0.99, 1.00)	1	13887
σ_0	Standard deviation of error distribution	1.20 (1.01, 1.83)	1	7627

Table S10 estimated unadjusted beta-binomial model parameters of the association between the SARS-CoV-2 and Influenza A index viral decline time and secondary transmission.

Parameter	Parameter description	Median (95% CrI)	$\widehat{R}$	n_{eff}
SARS-CoV-2				
ξ	Correlation with VL decline time	0.68 (-0.13, 1.77)	1	4167
z	Offset	-0.28 (-0.92, 0.39)	1	11212
θ	Beta distribution dispersion parameter	1.23 (0.34, 4.15)	1	12942
Influenza A				
ξ	Correlation with VL decline time	0.25 (-0.14, 0.63)	1	9366
z	Offset	-2.08 (-2.44, -1.76)	1	16725
θ	Beta distribution dispersion parameter	3.94 (2.03, 7.65)	1	19496

Table S11 estimated adjusted beta-binomial model parameters of the association between the SARS-CoV-2 and Influenza A index viral decline time and secondary transmission.

Parameter	Parameter description	Median (95% CrI)	$\widehat{R}$	n_{eff}
SARS-CoV-2				
ξ	Correlation with VL decline time	0.36 (-0.41, 1.39)	1	8668
γ_1	Index age adjustment	-1.68 (-4.30, 0.85)	1	10268
γ_2	Index age adjustment	-0.43 (-2.83, 1.92)	1	8851
γ_3	Index age adjustment	2.25 (-0.15, 4.67)	1	9289
κ	Household crowding parameter	-0.52 (-2.18, 1.02)	1	13561
θ	Beta distribution dispersion parameter	1.57 (0.44, 4.78)	1	18891
δ	Index chronic disease adjustment	-2.10 (-3.94, -0.37)	1	19047
z_1	Pre-alpha variant adjustment	-0.93 (-4.00, 1.98)	1	8729
z_2	Alpha variant adjustment	0.30 (-2.52, 3.07)	1	8274
z_3	Delta variant adjustment	1.48 (-1.90, 5.02)	1	8768
Influenza A				
ξ	Correlation with VL decline time	0.35 (-0.11, 0.79)	1	7370
γ_1	Index age adjustment	0.13 (-2.01, 2.37)	1	5235
γ_2	Index age adjustment	-2.35 (-5.34, 0.26)	1	9928
γ_3	Index age adjustment	0.77 (-1.43, 3.07)	1	5413
λ	Antiviral adjustment	-0.29 (-1.00, 0.40)	1	21849
θ_{vacc}	Beta distribution dispersion parameter (vaccinated contacts)	1.61 (1.02, 4.47)	1	19524
θ_{unvacc}	Beta distribution dispersion parameter (unvaccinated contacts)	4.42 (2.17, 8.43)	1	24007
v^{con}	Contact vaccination adjustment	-0.08 (-1.01, 0.96)	1	16108
v^{ind}	Index vaccination adjustment	0.04 (-0.91, 0.90)	1	16087
δ	Index chronic disease adjustment	-0.87 (-2.26, 0.24)	1	19303
z_1	Seasonal H1N1 adjustment	-2.22 (-4.58, 0.07)	1	5109
z_2	Seasonal H3N2 adjustment	-1.76 (-4.09, 0.54)	1	5032
z_3	Pandemic H1N1 adjustment	-1.99 (-4.64, 0.46)	1	5471

Table S12 Model evaluation using Leave One Out Cross Validation (LOO-CV). For each virus, the correlation between SAR and RNA decline time was modelled with (adjusted) or without (unadjusted) confounders. For SARS-CoV-2, the unadjusted model was found to be more predictive than the adjusted model, whereas for influenza adjusting for confounders produced a more predictive model. In both cases, however, the difference between the two models was relatively small. ELPD = expected log pointwise predictive density, for other definitions and method see reference [17]. Analysis used the R package loo [18].

Model	Difference	Standard error of difference	LOO estimate of ELPD	Standard error of LOO estimate of ELPD	Mean estimate of effective number of parameters	Standard error in estimate of effective number of parameters	Proportion of observations with Pareto k diagnostic values >=0.7
**SARS-CoV-2**							
Unadjusted	-6.4	4.0	-38	2.9	6.5	1.0	7.9%
Adjusted	0.0	0.0	-31.6	4.8	8.5	2.0	2.6%
**Influenza A**							
Unadjusted	0.0	0.0	-110.5	9.8	3.7	0.4	0 %
Adjusted	-9.3	2.8	-119.8	11.2	13.3	2.2	2.6 %

Table S13 estimated unadjusted logistic model parameters of the association between the SARS-CoV-2 index viral decline time and secondary transmission.

Parameter	Parameter description	Median (95% CrI)	$\hat{R}$	n_{eff}
SARS-CoV-2				
ξ	Correlation with VL decline time	0.78 (0.06, 1.96)	1	3862
z	Offset	-0.40 (-0.96, 0.16)	1	11803
Influenza A				
ξ	Correlation with VL decline time	0.26 (-0.10, 0.62)	1	8161
z	Offset	-2.19 (-2.53, -1.89)	1	16492

Table S14 estimated adjusted logistic model parameters of the association between the SARS-CoV-2 and Influenza A index viral decline time and secondary transmission.

**Parameter**	**Parameter description**	**Median (95% CrI)**	$\hat{R}$	n_{eff}
SARS-CoV-2				
ξ	Correlation with VL decline time	0.78 (-0.33, 2.33)	1	5123
γ_1^{ind}	Index age adjustment	-1.82 (-4.50, 0.73)	1	7350
γ_2^{ind}	Index age adjustment	-0.69 (-3.32, 1.78)	1	7812
γ_3^{ind}	Index age adjustment	2.67 (0.15, 5.26)	1	7572
γ_1^{con}	Contact age adjustment	-0.33 (-2.86, 2.21)	1	7436
γ_2^{con}	Contact age adjustment	0.21 (-2.23, 2.75)	1	6732
γ_3^{con}	Contact age adjustment	0.27 (-2.15, 2.69)	1	6778
δ^{con}	Contact chronic disease adjustment	-2.77 (-5.16, -0.67)	1	14566
δ^{ind}	Index chronic disease adjustment	-1.26 (-3.80, 0.94)	1	11820
κ	Household crowding adjustment	-0.75 (-2.65, 1.01)	1	8557
z_1	Pre-alpha variant adjustment	-0.84 (-4.40, 2.59)	1	4404
z_2	Alpha variant adjustment	-0.00 (-3.39, 3.33)	1	4275
z_3	Delta variant adjustment	1.91 (-1.91, 6.06)	1	5309
Influenza A				
ξ	Correlation with VL decline time	0.35 (-0.11, 0.79)	1	7370
γ_1^{ind}	Index age adjustment	0.13 (-2.01, 2.37)	1	5235
γ_2^{ind}	Index age adjustment	-2.35 (-5.34, 0.26)	1	9928
γ_3^{ind}	Index age adjustment	0.77 (-1.43, 3.07)	1	5413
γ_1^{con}	Contact age adjustment	-0.29 (-1.00, 0.40)	1	21849
γ_2^{con}	Contact age adjustment	1.61 (1.02, 4.47)	1	19524
γ_3^{con}	Contact age adjustment	4.42 (2.17, 8.43)	1	24007
λ	Index antiviral adjustment	-0.08 (-1.01, 0.96)	1	16108
v^{con}	Contact vaccination adjustment	0.04 (-0.91, 0.90)	1	16087
v^{ind}	Index vaccination adjustment	-0.87 (-2.26, 0.24)	1	19303
δ^{ind}	Index chronic disease adjustment	-2.22 (-4.58, 0.07)	1	5109
δ^{con}	Contact chronic disease adjustment	-1.76 (-4.09, 0.54)	1	5032
z_1	Seasonal H1N1 adjustment	-1.99 (-4.64, 0.46)	1	5471
z_2	Seasonal H3N2 adjustment	0.35 (-0.11, 0.79)	1	7370
z_3	Pandemic H1N1 adjustment	0.13 (-2.01, 2.37)	1	5235

Supplementary Materials References

[1] Rihn SJ, Merits A, Bakshi S, Turnbull ML, Wickenhagen A, Alexander AJT, et al. A plasmid DNA-launched SARS-CoV-2 reverse genetics system and coronavirus toolkit for COVID-19 research. PLoS Biol 2021;19:e3001091. https://doi.org/10.1371/journal.pbio.3001091.

[2] Rowan AG, May P, Badhan A, Herrera C, Watber P, Penn R, et al. Optimized protocol for a quantitative SARS-CoV-2 duplex RT-qPCR assay with internal human sample sufficiency control. Journal of Virological Methods 2021;294:114174. https://doi.org/10.1016/j.jviromet.2021.114174.

[3] Wolters F, Coolen JPM, Tostmann A, van Groningen LFJ, Bleeker-Rovers CP, Tan ECTH, et al. Novel SARS-CoV-2 Whole-genome sequencing technique using Reverse Complement PCR enables easy, fast and accurate outbreak analysis in hospital and community settings. Microbiology; 2020. https://doi.org/10.1101/2020.10.29.360578.

[4] Di Tommaso P, Chatzou M, Floden EW, Barja PP, Palumbo E, Notredame C. Nextflow enables reproducible computational workflows. Nat Biotechnol 2017;35:316–9. https://doi.org/10.1038/nbt.3820.

[5] Chen S, Zhou Y, Chen Y, Gu J. fastp: an ultra-fast all-in-one FASTQ preprocessor. Bioinformatics 2018;34:i884–90. https://doi.org/10.1093/bioinformatics/bty560.

[6] Li H. Aligning sequence reads, clone sequences and assembly contigs with BWA-MEM 2013.

[7] Danecek P, Bonfield JK, Liddle J, Marshall J, Ohan V, Pollard MO, et al. Twelve years of SAMtools and BCFtools. GigaScience 2021;10:giab008. https://doi.org/10.1093/gigascience/giab008.

[8] Wilm A, Aw PPK, Bertrand D, Yeo GHT, Ong SH, Wong CH, et al. LoFreq: a sequence-quality aware, ultra-sensitive variant caller for uncovering cell-population heterogeneity from high-throughput sequencing datasets. Nucleic Acids Research 2012;40:11189–201. https://doi.org/10.1093/nar/gks918.

[9] Pedersen BS, Quinlan AR. Mosdepth: quick coverage calculation for genomes and exomes. Bioinformatics 2018;34:867–8. https://doi.org/10.1093/bioinformatics/btx699.

[10] Cingolani P, Platts A, Wang LL, Coon M, Nguyen T, Wang L, et al. A program for annotating and predicting the effects of single nucleotide polymorphisms, SnpEff: SNPs in the genome of Drosophila melanogaster strain w ^{1118}; iso-2; iso-3. Fly 2012;6:80–92. https://doi.org/10.4161/fly.19695.

[11] Ewels P, Magnusson M, Lundin S, Käller M. MultiQC: summarize analysis results for multiple tools and samples in a single report. Bioinformatics 2016;32:3047–8. https://doi.org/10.1093/bioinformatics/btw354.

[12] Rambaut A, Holmes EC, O'Toole Á, Hill V, McCrone JT, Ruis C, et al. A dynamic nomenclature proposal for SARS-CoV-2 lineages to assist genomic epidemiology. Nat Microbiol 2020;5:1403–7. https://doi.org/10.1038/s41564-020-0770-5.

[13] O'Toole Á, Scher E, Underwood A, Jackson B, Hill V, McCrone JT, et al. Assignment of Epidemiological Lineages in an Emerging Pandemic Using the Pangolin Tool. Virus Evolution 2021:veab064. https://doi.org/10.1093/ve/veab064.

[14] Singanayagam A, Hakki S, Dunning J, Madon KJ, Crone MA, Koycheva A, et al. Community transmission and viral load kinetics of the SARS-CoV-2 delta (B.1.617.2) variant in vaccinated and unvaccinated individuals in the UK: a prospective, longitudinal, cohort study. The Lancet Infectious Diseases 2021:S1473309921006484. https://doi.org/10.1016/S1473-3099(21)00648-4.

[15] R Core Team. R: A Language and Environment for Statistical Computing 2022.

[16] Stan Development Team. RStan: the R interface to Stan. R package version 2.21.3. n.d.

[17] Vehtari A, Gelman A, Gabry J. Practical Bayesian model evaluation using leave-one-out cross-validation and WAIC. Stat Comput 2017;27:1413–32. https://doi.org/10.1007/s11222-016-9696-4.

[18] Vehtari A, G.J.Magnusson M, Yao Y, Bürkner P, Paananen TG. loo: Efficient leave-one-out crossvalidation and WAIC for Bayesian models:R package version 2.6.0 n.d.
